# Genome-Wide Association Study of Copy Number Variants Suggests *LTBP1* and *FGD4* Are Important for Alcohol Drinking

**DOI:** 10.1371/journal.pone.0030860

**Published:** 2012-01-25

**Authors:** Yu-Fang Pei, Lei Zhang, Tie-Lin Yang, Yingying Han, Rong Hai, Shu Ran, Qing Tian, Hui Shen, Jian Li, Xue-Zhen Zhu, Xingguang Luo, Hong-Wen Deng

**Affiliations:** 1 Center of System Biomedical Sciences, University of Shanghai for Science and Technology, Shanghai, People's Republic of China; 2 School of Life Science and Technology, Xi'an Jiaotong University, Xi'an, People's Republic of China; 3 The Affiliated Hospital of Inner Mongolia Medical College Huhhot, Inner Mongolia, People's Republic of China; 4 Department of Biostatistics, Tulane University, New Orleans, Louisiana, United States of America; 5 Department of Psychiatry, Yale University School of Medicine, New Haven, Connecticut, United States of America; 6 Laboratory of Molecular and Statistical Genetics and Key Laboratory of Protein Chemistry and Developmental Biology of Ministry of Education, College of Life Sciences, Hunan Normal University, Changsha, People's Republic of China; FuWai Hospital, Chinese Academy of Medical Sciences, China

## Abstract

Alcohol dependence (AD) is a complex disorder characterized by psychiatric and physiological dependence on alcohol. AD is reflected by regular alcohol drinking, which is highly inheritable. In this study, to identify susceptibility genes associated with alcohol drinking, we performed a genome-wide association study of copy number variants (CNVs) in 2,286 Caucasian subjects with Affymetrix SNP6.0 genotyping array. We replicated our findings in 1,627 Chinese subjects with the same genotyping array. We identified two CNVs, CNV207 (combined p-value 1.91E-03) and CNV1836 (combined p-value 3.05E-03) that were associated with alcohol drinking. CNV207 and CNV1836 are located at the downstream of genes *LTBP1* (870 kb) and *FGD4* (400 kb), respectively. *LTBP1*, by interacting *TGFB1*, may down-regulate enzymes directly participating in alcohol metabolism. *FGD4* plays a role in clustering and trafficking GABA_A_ receptor and subsequently influence alcohol drinking through activating *CDC42*. Our results provide suggestive evidence that the newly identified CNV regions and relevant genes may contribute to the genetic mechanism of alcohol dependence.

## Introduction

Alcohol is the most widely used addictive substance, affecting billions of people worldwide. Alcohol dependence (AD) is characterized by psychiatric and physiological dependence (withdrawal and tolerance) as well as behavioral indices of addiction on alcohol [1]. It is a common complex disease determined by both genetic and environmental factors; the estimated heritability through family and twin and adoption studies [2], [3], [4] is as high as 40 to 60%. Therefore, the identification of genes that contribute to AD variation will improve our understanding of genetic mechanism that underlies this disorder.

Regular drinking is necessary for the development of AD. The heaviness of alcohol consumption including the frequency and amount of alcohol drinking is strongly associated with AD risk. Alcohol consumption is largely determined by genetic factors. Most recently, at least two genome-wide association studies (GWASs) have identified *TMEM108*, *ANKS1A* and *AUTS2* as possible risk genes for alcohol consumption in European-origin populations [5], [6]. Recent studies have also demonstrated that there is a high degree of genetic overlap between AD and heaviness of consumption even among non-dependent individuals in the general population [7]. Genes affecting alcohol intake also affect AD risk [8]. Therefore, genetic study on alcohol drinking will likely have a useful role in the identification of genes contributing to AD.

Genome-wide linkage study (GWLS) have identified a number of loci potentially linked to AD [9], [10], [11], [12]. Association studies have also implicated several associated genes, such as *ALDH2*, *GABRA2*, *DRD2* [13], [14], [15]. However, the identified genes could only account for a small proportion of genetic variation, leaving the vast majority of heritability unknown. The limitation of traditional association studies is that they focus on single nucleotide polymorphisms (SNPs) only, while the human genome exhibits various types of genomic variation. The “missing” heritability may be hidden in these extensive types of variants.

Recent studies have shown that another type of genomic variation, copy number variations (CNVs), plays a significant role in influencing common diseases as well [16], [17]. CNV is a DNA segment that is one kilo bases or larger and presents at variable copy numbers in comparison with reference genome [18]. It covers approximately 12% (∼360 Mb) of the human genome [19]. CNVs may influence gene expression, phenotypic variation and adaptation by disrupting genes and altering gene dosage, and can cause diseases [20], [21], [22], [23]. Furthermore, CNVs can influence gene expression indirectly through position effects, predispose to deleterious genetic changes, or provide substrates for chromosome change in evolution [18], [24], [25], [26]. Many diseases are found to be associated with copy number changes, including schizophrenia [27], [28], Parkinson's disease [29], autism [30] and HIV/AIDS susceptibility [31]. Therefore, investigation of CNVs would contribute to unravel the genetic basis of complex diseases and phenotypes.

In this study, to uncover the genetic basis of alcohol drinking caused by CNVs, we conducted a genome-wide association study in 2,286 Caucasian subjects with Affymetrix SNP6.0 genotyping array. We further replicated our findings in 1,627 Chinese subjects.

## Materials and Methods

### Ethics Statement

The study was approved by Institutional Review Boards of Creighton University, University of Missouri-Kansas City, Hunan Normal University of China and Xi'an Jiaotong University of China. Signed informed-consent documents were obtained from all study participants before they entered the study.

### Subjects

#### 1) Discovery sample

The discovery sample consisted of 2,286 unrelated Caucasian subjects that were recruited in Midwestern US in Kansas City, Missouri and Omaha, Nebraska. All identified subjects were of European origin. Subjects with certain conditions were excluded, including chronic disorders involving vital organs (heart, lung, liver, kidney, brain), serious metabolic diseases (diabetes, hypo- and hyper-parathyroidism, hyperthyroidism, etc.), skeletal diseases (Paget disease, osteogenesis imperfecta, rheumatoid arthritis, etc.), chronic use of drugs affecting bone metabolism (hormone replacement therapy, corticosteroid therapy, anti-convulsant drugs), and malnutrition conditions (such as chronic diarrhea, chronic ulcerative colitis, etc.).

#### 2) Replication sample

To replicate the associations for CNVs at significant level of p<0.05, we performed another independent study in 1,627 unrelated subjects of Chinese Han population. All subjects were recruited from the cities of Xi'an/Changsha and their neighboring areas in China.

### Phenotyping

In Diagnostic Interview for Genetic Studies (DIGS) [32], they defined “Regular Drinking” as drinking at least once a week. With 6,006 Caucasian subjects from 1,131 pedigrees, we confirmed that this kind of “Regular Drinking” was genetically determined and the heritability estimation was ∼40% (unpublished data). So we adopted “Regular Drinking” as phenotype and considered subjects with drinking at least once per week as cases and subjects with drinking less than once per week as controls. For alcohol drinkers, alcohol consumption information was also collected. Alcohol consumption is graded as 1, 2, 3 and 4, representing drinking alcohol 1–2, 3–6, 7–10 and >10 times per week, respectively.

### Genotyping

Genomic DNA was extracted from peripheral blood leukocytes using standard protocols. Genotyping with Affymetrix Genome-Wide Human SNP Array 6.0, which features 1.8 million genetic markers, including more than 906,600 SNPs and more than 946,000 probes for detection of copy number variation, was performed using the standard protocol recommended by the manufacturer. Fluorescence intensities were quantified using an Affymetrix array scanner 30007 G. Data management and analyses were performed using the Affymetrix® GeneChip® Command Console® Software (AGCC). Contrast quality control (QC) threshold was set at the default value of greater than 0.4 for sample quality control. The final average contrast QC across the entire sample reached the high level of 2.32. The Birdsuite package [33] was used for genotype calling, genotyping quality control, and CNV identification.

For the discovery sample, common CNVs were identified using the CANARY algorithm implemented in the Birdsuite software [33], which utilized a previously defined copy number variation map based on HapMap samples. In total, 1,316 CNVs were genotyped for all the subjects in the discovery sample.

In order to generate results with high confidence, we conducted quality control (QC) filtering both at the sample level and the CNV level. First, for the sample level QC, we used three quality metrics reported by the Birdsuite method to evaluate the initial 2,286 subjects for quality in copy number genotyping. The following procedures were adopted: 1) we removed any sample that was greater or less than three standard deviations (SD) from the average estimate of copy number, which was approximate two copies at genome-wide level; 2) we calculated the variability in copy number and SNP probe intensities with each standardized per chromosome. We removed any sample with more than three SDs than these estimates on the average genome-wide level; 3) we removed any sample in which more than two chromosomes failed any of these three metrics, i.e. more than three SD in estimated copy number or excessive CNV or SNP variability for chromosome. According to above criteria, 71 subjects were discarded, the copy number of the remaining 2,215 subjects including 938 cases and 1,277 controls were successfully genotyped by the CANARY software.

Second, we conducted QC filtering at the CNV level. Out of the initial 1,316 CNVs, we discarded the CNVs with confidence score greater than 10% or with less than 1% of allele frequency (AF) that refers to the total proportion of the subjects with copy number less or more than two in total sample. Of the initial full-set of 1,316 CNVs, there are 501 CNVs with AF<1%, 268 CNVs with confidence score greater than 0.10 and 4 CNVs with both. As a result, 551 CNVs were available for subsequent association analyses.

### Statistical analyses

Statistical analyses of CNV-based association were performed by a logistic regression model in R package *glm* [34]. The copy number was used as a predictor of alcohol drinking. Age and sex were included as covariates in the model. To correct for the effect of potential population stratification, we performed a principal component analysis on genome-wide SNP data with EIGENSTRAT [35] and included the top ten principal components as covariates. After correcting for the effects of all covariates, we also exponentiated the coefficients for the CNVs and interpreted them as odds ratios (ORs). Fisher's method [36] was used to combine the p-values from both the discovery and replication samples.

We further tested the association of alcohol drinking and SNPs that locate in CNV regions. The association was tested by a logistic regression model.

## Results

The basic characteristics of the subjects used in both discovery and replication samples, including sex, age, number of subjects with regular alcohol drinking and drinking consumption are summarized in Table 1.

**Table 1:** Basic characteristics of the study subjects.

	Discovery Sample			Replication Sample		
	Total	Male	Female	Total	Male	Female
No. of subjects	2286	558	1728	1627	802	825
Age	51.37(13.75)	50.71(16.05)	51.58(12.92)	34.49(13.24)	31.43(7.97)	37.46(13.77)
No. of alcohol drinkers	972	296	676	167	137	30
Drinking consumption	2.14(1.00)	2.51(1.15)	1.98(0.88)	-	-	-

Note: age and drinking consumption are presented as mean (standard deviation).

In the discovery sample, 30 CNVs were associated with regular alcohol drinking at the significance level 0.05 (Table S1). Two of them, CNV207 and CNV1836, were further replicated in the replication sample. The p-values of CNV207 in the discovery and replication samples were 2.27E-02 and 8.87E-03; and those of CNV1836 were 8.13E-03 and 4.17E-02, respectively (Table 2). The odds ratios (ORs) of CNV207 in the discovery and replication samples were 1.16 (95% CI: 1.02–1.32) and 0.40 (95% CI: 0.21–0.82), respectively and those of CNV1836 in the discovery and replication samples were 1.22 (95% CI: 1.05–1.42) and 1.62 (95% CI: 1.02–2.72), respectively. The combined p-values of these two CNVs were 1.91E-03 and 3.05E-03, respectively. Though neither of the combined p-values achieved Bonferroni corrected significance level (9.07E-5), they offered suggestive signals of potential associations.

**Table 2:** Characteristics of the interesting CNVs for association analysis.

Name	Chr	Start	End	Discovery Sample				Replication Sample				Combined P-value
				P-value	AF	CS^a^	OR(95% CI)	P-value	AF	CS^a^	OR(95% CI)	
CNV1836	12	33,192,673	33,198,641	8.13E-03	0.38	0.005	1.22(1.05–1.42)	4.17E-02	0.24	0.028	1.62(1.02–2.72)	3.05E-03
CNV207	2	34,552,819	34,590,561	2.27E-02	0.63	0.003	1.16(1.02–1.32)	8.87E-03	0.07	0.011	0.40(0.21–0.82)	1.91E-03

a . CS denotes confidence score.

**Figure 1:**
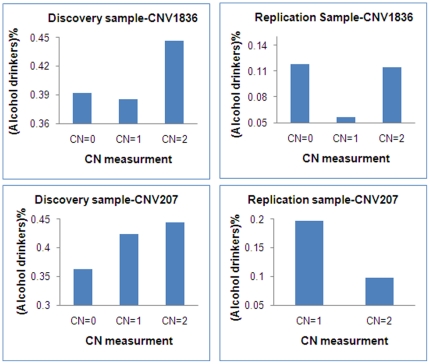
Percentage of alcohol drinkers in groups with different copy number (CN) of CNV207 and CNV1836 in the discovery and replication samples.

CNV207 is located in the chromosome region 2p22 with physical position ranging from 34,552,819 bp to 34,590,561 bp. It is in the 870 kb downstream of the gene transforming growth factor beta-1 binding protein 1(*LTBP1*). The numbers of carriers with 0, 1 and 2 copies were 287, 1087 and 810 in the discovery sample and 0, 71 and 1220 in the replication sample, respectively. Individuals with more CNs trended to have higher percentage of alcohol drinkers in the discovery sample, but individuals with more CNs trended to have lower percentage of alcohol drinkers in the replication sample (Figure 1).

CNV1836 is located in the chromosome region 12p11 with physical position ranging from 33,192,673 bp to 33,198,641 bp. It is in the 400 kb downstream of the gene actin-filament binding protein frabin (*FGD4*). The number of carriers of 0, 1 and 2 copy numbers was 102, 729 and 1353 in the discovery sample and 17, 264 and 1011 in the replication sample, respectively. Subjects with 1 copy number had the lowest percentage of alcohol drinkers in both discovery and replication samples (Figure 1).

There are two SNPs that are located in the region of CNV207 (*rs7593687* and *rs7583665*) and four in the region of CNV1836 (*rs10772040*, *rs983985*, *rs7972327* and *rs2018498*). None of these six SNPs was significantly associated with alcohol drinking in both samples (Table 3).

**Table 3:** Association between SNPs located in CNV207 and CNV1836 region and alcohol drinking.

CNV region	CHR	SNP	Discovery sample			Replication sample		
			Allele^a^	MAF	P-value^b^	Allele^a^	MAF	P-value^b^
CNV207	2	*rs7593687*	A/G	<0.01	NA	A/G	<0.01	NA
	2	*rs7583665*	T/C	0.02	0.75	T/C	<0.01	NA
CNV1836	12	*rs10772040*	A/C	0.09	0.88	C/A	0.22	0.08
	12	*rs983985*	C/T	0.37	0.19	T/C	0.04	0.16
	12	*rs7972327*	C/G	0.09	0.99	G/C	0.12	0.97
	12	*rs2018498*	A/G	0.37	0.19	G/A	0.04	0.16

a . The second allele is the minor allele in the sample;

b . NA: not available due to the low minor allele frequency.

## Discussion

In this study, we have performed a CNV-based genome-wide association study of alcohol drinking in 2,286 Caucasian subjects, and replicated in 1,627 Chinese subjects. We identified CNV207 and CNV1836 to be suggestively associated with alcohol drinking in both discovery and replication samples. Although the Bonferroni corrected p-values for CNV207 and CNV1836 are not significant, they were replicated in distinct populations and might be more generalizable to other populations, and would be more likely to be causal in nature [37].

*LTBP1* plays a critical role in controlling and directing the activity of transforming growth factor beta 1 (*TGFB1*) [38], which encodes a member of the transforming growth factor beta (*TGFB*) family. *TGFB* modulates the expression of genes involved in a variety of biological process, including alcohol metabolism and oxidative stress. In previous studies, Saltzman et al. [39] reported that alcohol drinking was associated with serum *TGFB1* levels. Ciuclan et al. [40] found that enzymes directly participating in alcohol metabolism were down-regulated by *TGFB*. *TGFB* was also found to strongly promote alcohol dependent oxidative stress and anti-oxidant depression leading to enhanced cellular toxicity [40]. This “alcohol damage promoting” effect of *TGFB* was further supported by the finding that ethanol induced lipid peroxidation was increased upon parallel activation of its signaling pathway [40]. Therefore *LTBP1* may influence alcohol drinking through its regulation effect on *TGFB* family.

Another relevant gene is *FGD4*. It activates the gene *CDC42*, which has a specific role in clustering and trafficking of *GABA_A_* receptors [41], [42]. There is a large body of evidence showing that *GABA_A_* receptors play central roles in both short- and long-term effects of ethanol in the central nervous system [43]. Therefore, *FGD4* gene may play a role in clustering and trafficking *GABA_A_* receptor through activating *CDC42* and subsequently influence alcohol drinking.

It is not surprising that the two related genes are not located in the respective CNV regions. A previous genome-wide association study between CNVs and gene expression levels showed that more than half of the associated genes were not located in any CNV interval [44]. These observations suggest that rather than altering gene dosage, CNV could also affect regulatory regions and other functional regions that have an impact on gene expression [44]. Stranger et al. [45], [46] showed that genes could be implied from CNVs as long as 2 MB apart, as confirmed by extensive studies in animal models [47], [48].

Notice that for CNV207, the association directions in the discovery and replication studies were not consistent. This inconsistency may be explained by the following reasons: first, genetic variants could have differences, such as different allele frequencies among diverse populations because of different evolution histories, resulting in different modes of genotype-phenotype association [49]. According to a recent study, replicable findings in distinct populations might be more generalizable to other populations, and would be more likely to be causal in nature [37]. Second, in the context of GWAS, significant associations are usually declared at genetic markers that are in linkage disequilibrium with causal site, rather than the causal site itself. Therefore, the directional inconsistency could be a result of opposite patterns of LD between the two populations.

We did not identify any significant SNPs within the two CNV regions, implying that CNV may take the effect alone.

In summary, we have indentified two CNVs, CNV207 and CNV1836, that were suggestively associated with alcohol drinking. The two relevant genes, *LTBP1* and *FGD4*, may play important roles in the metabolism of alcohol dependence. Our findings may provide informative candidate genes for further functional studies of alcohol dependent traits.

## Supporting Information

Table S1 Information of 30 CNVs with p<0.05 in discovery sample.
(DOC)
