# Supplementary material for: Genome-Wide Association Study of Copy Number Variants Suggests LTBP1 and FGD4 Are Important for Alcohol Drinking
Source: PLoS One. 2012 Jan 25;7(1):e30860. doi: 10.1371/journal.pone.0030860 (PMC3266269; doi:10.1371/journal.pone.0030860)
Supplement: Table S1 — Information of 30 CNVs with p<0.05 in discovery sample. (DOC) [file pone.0030860.s001.doc]

Table S1. Information of 30 CNVs with p<0.05 in discovery sample.

| Name | Chr | Start | End | Discovery Sample | | | Replication Sample | | | Combined P-value^b^ |
| --- | --- | --- | --- | --- | --- | --- | --- | --- | --- | --- |
|  |  |  |  | P-value | AF | CS^a^ | P-value^b^ | AF | CS^a^ |  |
| CNV11019 | 6 | 8872510 | 8878099 | 1.77E-03 | 0.02 | 0.015 | NA | 0.02 | 0.042 | NA |
| CNV1760 | 11 | 85567306 | 85576469 | 4.69E-03 | 0.04 | 0.024 | NA | 0.02 | 0.015 | NA |
| CNV1836 | 12 | 33192673 | 33198641 | 8.13E-03 | 0.38 | 0.005 | 4.17E-02 | 0.24 | 0.028 | 3.05E-03 |
| CNV12319 | 15 | 41680133 | 41682098 | 1.08E-02 | 0.05 | 0.090 | 7.01E-01 | 0.07 | 0.110 | 4.46E-02 |
| CNV2246 | 17 | 19440328 | 19479039 | 1.13E-02 | 0.02 | 0.004 | 7.92E-01 | 0.03 | 0.019 | 5.13E-02 |
| CNV10767 | 4 | 104961839 | 104980713 | 1.20E-02 | 0.02 | 0.030 | 2.59E-01 | 0.01 | 0.056 | 2.10E-02 |
| CNV874 | 5 | 150185693 | 150198797 | 1.80E-02 | 0.17 | 0.017 | 9.69E-01 | 0.69 | 0.021 | 8.81E-02 |
| CNV2084 | 15 | 37375518 | 37379480 | 2.36E-02 | 0.09 | 0.092 | 2.14E-01 | 0.23 | 0.084 | 3.18E-02 |
| CNV2113 | 15 | 74678296 | 74682830 | 2.36E-02 | 0.75 | 0.044 | 3.98E-01 | 0.51 | 0.040 | 5.33E-02 |
| CNV2260 | 17 | 36786395 | 36790200 | 2.63E-02 | 0.02 | 0.074 | 1.09E-01 | 0.03 | 0.174 | 1.96E-02 |
| CNV207 | 2 | 34552819 | 34590561 | 2.27E-02 | 0.63 | 0.003 | 8.87E-03 | 0.07 | 0.011 | 1.91E-03 |
| CNV315 | 2 | 174301193 | 174305814 | 2.80E-02 | 0.03 | 0.088 | 4.99E-01 | 0.07 | 0.259 | 7.36E-02 |
| CNV10525 | 3 | 63108165 | 63110809 | 2.90E-02 | 0.01 | 0.070 | 7.11E-01 | 0.04 | 0.118 | 1.01E-01 |
| CNV12744 | 20 | 59003765 | 59023478 | 2.94E-02 | 0.03 | 0.005 | 3.52E-01 | 0.03 | 0.024 | 5.76E-02 |
| CNV248 | 2 | 87227404 | 87267503 | 2.98E-02 | 0.05 | 0.077 | 4.58E-01 | 0.03 | 0.010 | 7.23E-02 |
| CNV1343 | 8 | 112363455 | 112364436 | 3.03E-02 | 0.86 | 0.036 | 9.49E-01 | 0.34 | 0.070 | 1.31E-01 |
| CNV1879 | 12 | 98319424 | 98322865 | 3.07E-02 | 0.25 | 0.049 | 8.55E-01 | 0.02 | 0.023 | 1.22E-01 |
| CNV10324 | 2 | 87668332 | 87753000 | 3.09E-02 | 0.06 | 0.055 | 3.03E-01 | 0.04 | 0.033 | 5.32E-02 |
| CNV10177 | 1 | 187353623 | 187359115 | 3.15E-02 | 0.01 | 0.054 | 3.72E-01 | 0.03 | 0.086 | 6.38E-02 |
| CNV104 | 1 | 147303148 | 147526040 | 3.18E-02 | 0.28 | 0.032 | 3.29E-01 | 0.26 | 0.049 | 5.82E-02 |
| CNV10549 | 3 | 89477282 | 89502071 | 3.30E-02 | 0.10 | 0.000 | 8.69E-01 | 0.03 | 0.024 | 1.31E-01 |
| CNV12610 | 18 | 65358832 | 65368255 | 3.65E-02 | 0.10 | 0.044 | 7.35E-02 | 0.04 | 0.028 | 1.85E-02 |
| CNV10446 | 2 | 227051032 | 227054312 | 3.71E-02 | 0.04 | 0.054 | 7.73E-01 | 0.03 | 0.132 | 1.31E-01 |
| CNV1690 | 11 | 18905648 | 18918564 | 3.81E-02 | 0.47 | 0.055 | 9.94E-01 | 0.30 | 0.072 | 1.62E-01 |
| CNV1875 | 12 | 86055044 | 86057392 | 4.05E-02 | 0.02 | 0.053 | 1.99E-01 | 0.05 | 0.137 | 4.69E-02 |
| CNV84 | 1 | 108535758 | 108539019 | 4.37E-02 | 0.42 | 0.043 | 9.84E-01 | 0.71 | 0.050 | 1.78E-01 |
| CNV555 | 4 | 10001452 | 10009766 | 4.54E-02 | 0.45 | 0.002 | 5.49E-01 | 0.25 | 0.003 | 1.17E-01 |
| CNV148 | 1 | 195089940 | 195168372 | 4.59E-02 | 0.02 | 0.001 | 7.90E-01 | 0.06 | 0.009 | 1.57E-01 |
| CNV10501 | 3 | 30967806 | 30973753 | 4.75E-02 | 0.02 | 0.055 | 4.55E-01 | 0.03 | 0.184 | 1.05E-01 |
| CNV2184 | 16 | 54353890 | 54379945 | 4.94E-02 | 0.30 | 0.078 | 1.05E-01 | 0.66 | 0.017 | 3.25E-02 |

a. CS denotes confidence score;

b. NA: not available.
